# An assessment of mental health policy in Ghana, South Africa, Uganda and Zambia

**DOI:** 10.1186/1478-4505-9-17

**Published:** 2011-04-08

**Authors:** Edwige Faydi, Michelle Funk, Sharon Kleintjes, Angela Ofori-Atta, Joshua Ssbunnya, Jason Mwanza, Caroline Kim, Alan Flisher

**Affiliations:** 1 Mental Health Policy and Service Development, Department of Mental Health and Substance Abuse, World Health Organisation, Switzerland; 2 Department of Psychiatry and Mental Health, University of Cape Town, South Africa; 3 University of Ghana Medical School, Ghana; 4 Department of Mental Health and Community Psychology, Makere University, Uganda; 5 Sociology Division, Department of Social Development Studies, University of Zambia

## Abstract

**Background:**

Approximately half of the countries in the African Region had a mental health policy by 2005, but little is known about quality of mental health policies in Africa and globally. This paper reports the results of an assessment of the mental health policies of Ghana, South Africa, Uganda and Zambia.

**Methods:**

The WHO Mental Health Policy Checklist was used to evaluate the most current mental health policy in each country. Assessments were completed and reviewed by a specially constituted national committee as well as an independent WHO team. Results of each country evaluation were discussed until consensus was reached.

**Results:**

All four policies received a high level mandate. Each policy addressed community-based services, the integration of mental health into general health care, promotion of mental health and rehabilitation. Prevention was addressed in the South African and Ugandan policies only. Use of evidence for policy development varied considerably. Consultations were mainly held with the mental health sector. Only the Zambian policy presented a clear vision, while three of four countries spelt out values and principles, the need to establish a coordinating body for mental health, and to protect the human rights of people with mental health problems. None included all the basic elements of a policy, nor specified sources and levels of funding for implementation. Deinstitutionalisation and the provision of essential psychotropic medicines were insufficiently addressed. Advocacy, empowerment of users and families and intersectoral collaboration were inadequately addressed. Only Uganda sufficiently outlined a mental health information system, research and evaluation, while only Ghana comprehensively addressed human resources and training requirements. No country had an accompanying strategic mental health plan to allow the development and implementation of concrete strategies and activities.

**Conclusions:**

Six gaps which could impact on the policies' effect on countries' mental health systems were: lack of internal consistency of structure and content of policies, superficiality of key international concepts, lack of evidence on which to base policy directions, inadequate political support, poor integration of mental health policies within the overall national policy and legislative framework, and lack of financial specificity. Three strategies to address these concerns emerged, namely strengthening capacity of key stakeholders in public (mental) health and policy development, creation of a culture of inclusive and dynamic policy development, and coordinated action to optimize use of available resources.

## Background

Worldwide, there is a significant gap between the level of mental health needs and the availability of quality services to appropriately address these needs. In low- and middle-income countries (LMICs), in Africa as elsewhere, it is estimated that between 76% and 99% of people with serious mental disorders do not have access to the treatment they need for their mental health problems [1-3].

Mental health policies and plans are essential tools for setting strategic priorities, coordinating action and reducing fragmentation of services and resources. They are more likely to achieve the desired effect when they reflect a clear commitment from governments, are well conceptualized, are consistent with the existing evidence base and international standards, and reflect a broad consensus among key stakeholders.

**Figure 1:**
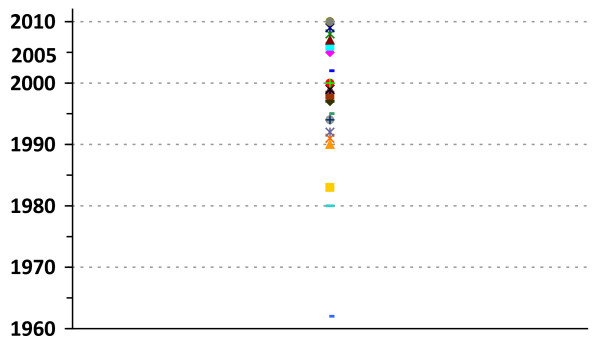
**Year of approval of mental health policies in countries of the WHO African Region**.

Approximately half the countries in the African Region had a mental health policy by 2005 [4]. There appears to have been an acceleration of policy development in Africa over the last five to ten years, as indicated in Figure 1, that may be linked to the recommendations of the World Health Report 2001 and the production and dissemination of technical information through the WHO Mental Health and Policy Service Guidance package [5]. However, very little is known about the quality of these mental health policies in Africa and globally, in terms of their content and the process followed in their development [6].

This paper reports the results of an assessment of the mental health policies of Ghana (*1994 mental health policy*), South Africa (*1997 mental health policy guidelines*), Uganda (*2000-2005 draft mental health policy*) and Zambia (*2005 mental health policy*), using the World Health Organization (WHO) Mental Health Policy Checklist. This assessment has been undertaken as part of the work of the Mental Health and Poverty Project (MHaPP). The MHaPP was an international research consortium (2005-2010) investigating the policy-based, legal and planning interventions required to break the cycle of poverty and mental illness in LMICs [7]. Members of the consortium elsewhere have provided a qualitative comparative analysis of stakeholder perceptions of barriers to and opportunities for improving the prioritisation, development and implementation of mental health policies within the 4 countries [8]. The current paper complements this work in deriving lessons for improving policy development and implementation based on the findings of a documentary analysis of the policy content and comprehensiveness of the policy development process in each of the countries.

## Methods

### The WHO Mental Health Policy Checklist

The WHO Mental Health Policy Checklist was developed by the WHO Department of Mental Health and Substance Abuse as a part of its Mental Health Policy and Service Guidance Package [9]. It was elaborated on the basis of a comprehensive review of the literature, consultation with policy makers and planners, and health professionals involved in policy and plan formulation and implementation as well as best practices in policy development from a range of low, middle, and high income countries [10]. The checklist sets out to assess whether key processes have been followed that are likely to lead to the successful adoption and implementation of the policy; and whether the content addresses certain critical issues such as protection of human rights, an evidence-based approach and the development of community-based care. It is divided into two sections. The first focuses on the process issues for policy development while the second addresses its structure and content. Each question has four response options: "yes/to a great degree", "to some extent", "no/not at all" or "unknown". For each item, additional space is provided for respondents to provide further contextual information and to detail the actions required to improve the policy and plan. The checklist is complimentary to the WHO framework for developing mental health policies and plans, a summary of which is provided in Table 1[11]. This paper describes the results of the checklist assessment and discusses the possible factors having influenced the development and final content of these policy documents, with suggestions for improvement.

**Table 1:** WHO Framework for the development of a mental health policy and plan (WHO, 2005)

Phase	Focus	Activities
**The mental health policy**		

1.	Information & data for policy development	Information is gathered through formal research and rapid appraisal to understand the mental health needs of the population and the mental health system and services offered.

2.	Evidence for effective strategies	Local services are visited and national and international literature reviewed (previous policy, plans and programmes, pilot projects and local experiences, evidence from countries or regions with similar cultural and socio-economic features).

3.	Consultation and negotiation	Consult with various stakeholders, draft proposals that blend different views with evidence from national and international experience, and available technical and resource base. Obtain political support for proposed policy.

4.	Exchange with other countries	Investigate the latest advances in more developed countries and lower-cost interventions in less developed countries, engage with international experts about proposals for the policy.

5.	Vision, values, principles and objectives	Set out the vision, values, principles and objectives for mental health in a draft policy. Objectives should focus on improving the health of the population, responding to people's expectations (human rights, client -focused orientation) and providing financial protection against the cost of ill-health.

6.	Areas for action	Translate the objectives of the mental health policy into areas for action, including those related to financing, legislation and human rights, organization of services, human resources and training, promotion, prevention, treatment and rehabilitation, essential drug procurement and distribution, advocacy, quality improvement, information systems, research and evaluation of policies and services and intersectoral collaboration.

7	Roles and responsibilities of different sectors	Consult governmental agencies (health, education, employment, social welfare, housing, justice); academic institutions; professional associations; general health and mental health workers; consumer and family groups; providers; nongovernmental organizations (NGOs); & traditional health workers.

**The mental health plan to support the policy**		

1.	Strategies & timeframes	Strategies and timeframes need to be set for the different areas of action identified in Step 6, through consultation with stakeholders.

2.	Indicators and targets	Strategies are broken down into specific targets and indicators to be achieved within given timeframes.

3.	Major activities	Activities and expected outputs to realize each strategy, and in each area of action, must be detailed.

4.	Costs, resources and budgets	Calculate the costs of each strategy, determine who will finance these resources, what resources are available resources and budget accordingly.

### WHO checklist evaluation in the 4 participating countries

#### Ghana

The *1994 Ghana Mental Health Policy *document was evaluated using the WHO Policy Checklist by a committee in Ghana comprising Ministry of Health officials, research investigators, and health and mental health professionals. Each member of the committee was provided with an electronic copy of the document and the checklist a few weeks prior to a scheduled committee meeting [12]. The committee then met and completed the WHO Policy Checklist together, over a period of two days. The review process was conducted item by item, with each item read aloud to the committee, after which members provided relevant anecdotal or documentary evidence for each item. Checklist evaluations were then done independently, followed by discussion of comments and the recording of a consensus evaluation. Any clarifications on unresolved issues were directed to the Chief Psychiatrist and the Chief Medical Officer in function at the time of drafting the policy, both of whom had been involved in the formulation of the policy.

#### Uganda

The Ugandan *Draft Mental Health Policy (2000-2005) *was initially evaluated during a stakeholders' workshop [13]. A total of 36 participants took part in the evaluation, and included representatives from various groups such as mental health professionals, non-governmental organisations (NGOs) dealing in mental health, workers from national and regional hospitals, the Makerere University Medical School, user groups and associations, the MHaPP research team, the Ministry of Health Policy Analysis Unit, and other government sectors and ministries. The WHO Policy Checklist was used to guide the evaluation. Participants were provided with a copy of the draft mental health policy document prior to the workshop. At the workshop, participants were divided into three subgroups: one subgroup discussed policy process issues, the second discussed policy content issues, and the third discussed human rights issues. Checklist evaluations were then pooled and recorded.

#### South Africa

South Africa's first post-apartheid national mental health policy guidelines was approved by departmental processes in 1997, the same year the Department of Health published the White Paper for the transformation of the health system in South Africa which includes a chapter on the development of mental health services at national, provincial, district and community levels [14]. The policy guidelines specifically direct that it should be read in conjunction with the provisions of the White Paper. The guidelines were not formally disseminated for implementation, and are not regarded as official policy by current health officials. The WHO Policy Checklist was completed for the *1997 Mental Health Policy Guidelines *and the *White paper on Health *together [15]. These two documents have been used by several provincial mental health program managers and at the national level to guide the development of programs, guidelines and standards in the past decade, and have therefore had an important impact on the development of mental health services and care in South Africa. The checklist was first completed by four members of the Cape Town MHaPP team, one of whom was a mental health programme manager responsible for implementing the policy at provincial level, and another involved in national mental health policy development and provincial mental health service delivery and management at the time that the policy was developed and in the ensuing decade thereafter. The policy was then reviewed by the former national Director of Mental Health and Substance Abuse, who had developed the policy guidelines. In addition, relevant information from the semi-structured interviews held with the nine provincial mental health coordinators for the country as part of MHaPP's broader situational analysis of mental health services in South Africa, informed the completion of the checklist. Comments from both reviews were integrated into a single consensus document.

#### Zambia

The final draft of the 2005 Zambia Mental Health Policy was evaluated using the WHO Policy Checklist by a committee of nine participants [16]. The participants were mainly staff from the Ministry of Health; three of them had been involved, as members of the technical committee, in the drafting of the mental health policy. In addition, the MHAPP country team independently assessed the policy document, using the same methodology.

#### Evaluation: WHO

Once each country had evaluated its most recent mental health policy using the WHO Policy Checklist, an independent team of staff members from WHO headquarters in Geneva with expertise in the area of mental health policy, planning and service development re-evaluated the policy using the WHO Policy Checklist, ensuring that responses to the checklist items were complete and consistent. The results of each evaluation were discussed until both the country-based committee and the WHO team were in agreement. This allowed for more objective evaluation across all policy checklist assessments.

## Results

### Mandate, level of approval and official dissemination

Respondents of the evaluation committee in all four countries reported that their mental health policy had received a high level mandate. However, the level of approval of the policy differed across countries. In Ghana, the *1994 mental health policy *was mandated and approved by the Director General of health services. The Minister of Health had nominated the chief psychiatrist at the time as advisor and had been kept involved all through the process. In South Africa, the *1997 mental health policy guidelines *were mandated by the Minister of Health and approved at a meeting of the Health Minister, the heads of health departments at provincial and national levels, and the nine provincial Members of Executive Councils (MECs). However, the 1997 *South Africa mental health policy guidelines *are not recognized as a formal policy by the current national Department of Health because they did not follow more recently adopted policy development protocols and were not formally published for dissemination. In Uganda, the *2000-2005 draft mental health policy*, although mandated by the Ministry of Health senior management, did not receive formal approval. However the policy was informally used to guide the Mental Health programme and activities nationwide. Finally, the *2005 Zambia mental health policy *was mandated and approved by Cabinet. In all cases the policy had not been sufficiently disseminated.

### Evidence base for policy development

The use of evidence for policy development varied considerably between countries. In Ghana, South Africa and Uganda no formal situational analysis or needs assessment was conducted. In Ghana, the policy was developed based on the personal experience of and information gathered through regional visits to psychiatric services by two senior health officials involved in psychiatric care and policy development in the country. Drafters in Uganda and South Africa also used their personal knowledge of the mental health situation in their countries, as well as unsystematic reviews of available small- scale local studies on the mental health situation in those countries to inform the policy process. In Zambia, the policy was informed by a four year situational analysis of the level of needs and services required in the country (1998-2002) conducted by the Ministry of Health in collaboration with an international consortium [17]. Data on general and mental health social and policy environment, health stewardship, mental health burden and stakeholder needs, and the human and financial resources both available and required were examined as part of the comprehensive country level profile. This information supplied drafters with key data needed to provide the necessary services at all levels of care in the country [18].

The director of mental health, actively involved in the development of the South Africa policy, was in contact with other countries such as Chile, Cuba and Zimbabwe in order to extract lessons learned for their policy development. Ghana, Zambia and Uganda reported having reviewed policies and experiences of other countries prior to drafting but did not report any type of formal exchange.

### Consultations

The majority of consultations were held within the mental health sector for all countries. In Zambia and Ghana, the health sector was consulted more broadly and only in South Africa were consultations extended to other ministries, such as those of social development and finance. Consultations, in general, were reported to be minimal in Uganda in comparison to the other countries. From a human rights perspective, it is interesting to note that Zambia was the only country that consulted service users. In South Africa, the policy drafters had expected the South African Federation for Mental Health, a national mental health non-governmental organization to provide service user input for policy development, however the input they provided was based solely on provider views.

### Vision, principles and objectives

Only the Zambian policy presented a clear vision. Values and principles underlying the policies are clearly spelt out in three of the four countries. The Ghanaian 1994 mental health policy was structured around a list of objectives or areas of actions. Both the Ugandan and Zambian policy documents had clearly defined objectives. In the case of Uganda these were consistent with the goal ('general objective'), values and principles whereas in the Zambian policy, objectives were only partly consistent with the 'guiding principles', the vision and the mission statement. For example, while the mission statement focused on the will "to provide all Zambians with equity of access to cost-effective, quality mental health care as close to the family as possible through use of comprehensive promotive, preventive, curative and rehabilitative mental health services", none of the five policy objectives listed covered curative aspects of treatment. The 1997 South African mental health policy guidelines did not set clear objectives.

Community care and integration of mental health care were the most strongly supported values by all MHaPP countries (see Table 2). The need for evidence-based practice was consistently acknowledged but only strongly in the Ugandan policy. Focus on human rights issues was relatively light, except for the Zambia policy, and was absent from the Ghana document. Intersectoral collaboration and social inclusion were mentioned by most countries but not as strongly as the other key values.

**Table 2:** Key values and principles promoted in the policies

Key values/country	Ghana	South Africa	Uganda	Zambia	*Total*	*Rank*
Integration of mental health into general health services	++	++	++	+	7	1

Community-based care	++	++	0	++	6	2

Evidence based practice	+	+	++	+	5	3

Human rights	0	+	+	++	4	4

Intersectoral collaboration	+	+	0	+	3	5

Social inclusion	+	+	+	0	3	6

Equity with physical health	0	0	0	0	0	7

Key:

0 - not at all addressed

+ - somewhat emphasized

++ - strongly emphasized

None of the MHaPP countries raised equity of mental health care with physical health care as a key value.

### Areas for Action

'Areas for action' referred to as 'key priority areas' in the South African policy, 'policy measures' in the Zambian policy or 'policy areas' in the Ghanaian policy were somewhat included in all policy documents reviewed. However, in the South African policy, 'key priority areas' were not sufficiently elaborated to give precise policy directions. In the Ugandan policy, policy directions in each of the main domains (e.g. human resources, essential medicines, information systems, human rights protection and promotion) were discussed together with broader concepts, values and principles under the 'guiding principles' section, blurring the boundaries between aspiration and actual commitment to action. In the Zambian policy, measures were quite comprehensively developed. The important interrelationships between the different areas for action were not described in any of the policies.

#### Coordination and management

Three of the four mental health policies stated the need to establish a coordinating position/body for mental health but only Ghana clearly specified membership, their terms of reference and functions. In the case of South Africa, coordination and management issues were addressed in the *South African White paper on Health *but not specifically mentioned in the *1997 mental health policy guidelines*. The White paper on Health was a document that set out the structure for reform of the health system in the post-apartheid era and included a chapter on mental health. It is meant to be read in conjunction with the *1997 mental health policy guidelines*.

#### Financing

All mental health policies did not systematically specify sources and levels of funding required to finance the implementation of the mental health policy. For example, financing is not addressed at all in the Ugandan policy. The Zambian policy broadly mentioned that the financing of mental health activities is related to the basic health care package and Sector Wide Approach but did not clearly present any financial sources and mechanisms to support implementation of the policy. The South African policy also noted the need to redistribute budget allocations, following the logic of the shift from institutional towards community-based services, but did not provide any further financial specifications in order to concretely support this policy shift.

#### Legislation and human rights

The need to promote and protect the human rights of people with mental health conditions is briefly referred to in three of the four policy documents and, in the case of South Africa, the *White Paper on Health *specifies a national responsibility to "review and evaluate legislation relating to mental health and substance abuse to safeguard the human rights of all service users". However, with the exception of Uganda, a significant number of key elements of a human rights approach such as accessibility, acceptability and affordability of care, equality, freedom from discrimination, involvement and empowerment of users and their families were omitted. Both the Zambian and the Ugandan policies highlighted the need to revise their current mental health legislation, with the latter clearly stipulating the need for monitoring mechanisms such as periodic reviews of the legislation and the establishment of a mental health board whose mandate included the investigation of complaints by patients.

#### Organization of services

A clear focus on community-based services can be found in all the mental health policies analysed, except for South Africa which did not spell out the community-oriented approach as strongly as the other countries did. Integration of mental health into general health care is also clearly highlighted in all countries, except for Zambia, with a particular focus on integration at the primary care level for Ghana and Uganda. Deinstitutionalisation is omitted in all four policies.

#### Promotion, prevention and rehabilitation

While promotion was mentioned in all four policies, prevention was clearly addressed only in the South African and the Ugandan policies. All policies mentioned rehabilitation, which was sufficiently elaborated in the case of Ghana and Uganda and to a lesser degree for South Africa and Zambia. Ghana had one policy section fully dedicated to 'rehabilitation of the mentally ill in the community'. It strongly stated the need to provide rehabilitation opportunities for persons with mental health conditions in their community, committed both the Ministry of Health and the Ministry of Social Welfare to provide community rehabilitation centres (day care centres, half way homes or hotels) and highlighted the collaboration needed between different relevant Ministries, the Mental Health Associations and NGOs in this policy area. The Ugandan policy provided a clear and comprehensive policy description of what will be done for rehabilitation of people with a mental health condition in the country. The policy listed the components expected to be included in programmes, and emphasized both the need to provide facilities and services as well as the need to encourage full employment and the selective placement of people with mental health conditions in employment. Both the South African and the Zambian policies mentioned in very broad terms the need for rehabilitation to assist people with mental health conditions but did not provide any further details on such interventions.

#### Essential psychotropic medicines

None of the four policies discussed procurement, distribution and storage of essential psychotropic medicines. The Ghana 1994 mental health policy called for the 'free treatment for the mentally ill' in its policy section 14, but remained totally silent on the strategies to achieve this objective. Each of the other three mental health policies included superficial statements on essential psychotropic medicines: while the Zambia policy committed to improve availability, it did not address accessibility; South Africa policy guidelines clearly stipulated the need to develop treatment protocols and to update the essential drugs list (EDL) but kept a very clinical focus on the use of medicines. Key issues about the distribution of psychotropic medicines directly to primary care and community centres as well as regulations about the type of health care workers able to prescribe and dispense medications were not addressed.

#### Advocacy

Advocacy in the mental health policies reviewed is generally limited to awareness raising campaigns and fundraising for mental health activities from other ministries and funding agencies. Empowerment of users and families is given variable attention. The South African and Ugandan policies mentioned the need to involve mental health users and families in different stages of the design, planning, implementation and evaluation of services. The Ghanaian policy remained weak in its intended support and empowerment of users' and families' organizations. The Zambia policy contained an ambiguous statement on the empowerment of users and their supporters which blurs the description of their roles, duties and rights.

#### Quality improvement

The need for quality improvement was quite strongly addressed in the Ugandan policy as quality assurance and evidence based services were guiding principles. The three other mental health policies mentioned the need for research on quality improvement (South Africa); quality assurance mechanisms and monitoring/evaluation of services (Ghana); or high quality evidence-based interventions and monitoring/evaluation of services (Zambia). None considered in a comprehensive manner the different aspects of quality improvement for mental health recommended by the WHO, such as alignment of policy for quality improvement, standards development, accreditation procedures, monitoring mechanisms, integration into management and delivery of services, reform of services and review of quality mechanisms [19].

#### Information systems

Information systems for mental health are not addressed by the South African or the Ghanaian mental health policies, while the Zambian policy makes a general statement about the need to develop mental health indictors within the general health information system. Only the Ugandan policy specifies the development of a mental health information system comprised of indicators embedded at each level of care to inform evidence based service development and defined roles and responsibility for its implementation.

#### Human resources and training

Human resources and training is one of the most important areas for action in a mental health policy, yet this issue was only comprehensively addressed in the Ghanaian policy where policy directions were provided for recruitment, training, working conditions, incentives and retention of health workers. The South African policy did not provide any further policy direction than stating the need to maintain a balance between psychiatric and other mental health services in the allocation of human and financial resources, and to develop specific competencies through district based heath worker training. The Zambian policy only addressed recruitment and training issues. The Ugandan policy outlined training issues for health and mental health professionals under the description of health services and facilities, level by level. However, as policy choices for human resources and training are diffused throughout the whole Ugandan policy document instead of being incorporated into one solid section, the information appears fragmented and the policy lacks clarity on this important issue.

#### Research and evaluation

While the Ugandan policy spells out a process for research and evaluation, in collaboration with a wide range of stakeholders (e.g. teaching institutions, NGOs, private and complementary practitioners, consumers) and provided details on the evaluation tools to be used, the three other policies assessed either did not address research and evaluation (Ghana) or did so only very broadly (South Africa, Zambia). None of the policies stipulated that policy and plans should also be monitored and evaluated. The Ugandan policy specified the development of a research agenda as a policy focus but does not elaborate on this. South Africa's policy noted the need for studies in mental health epidemiology and intervention effectiveness in the South African context, as well as specifying priority areas for research including prevention of substance abuse, violence prevention, the mental health of women, children and youth, and studies on the direct and indirect costs of mental illness in line with the broader national research agenda for the country. The Zambian policy restricted its mention of research to a need for research focused on epidemiology, to inform service development, for the evaluation of service delivery, and, as in the Ugandan policy, noted the development of a research agenda as a policy priority.

#### Intrasectoral and intersectoral collaborations

The Ghanaian, Ugandan and Zambian policies spoke in broad terms of the need for collaboration on mental health issues within the health sector while the South African policy focused its attention on traditional practitioners and the private health sector. However, none of the policies detailed the specific roles and responsibilities of the different partners, as well as the nature of the possible collaboration. Similarly, while some of the non-health sectors expected to be involved in mental health service development (e.g. education, justice, housing, labour) are mentioned to varying degrees in all the policies, none of these policies commit to the roles and responsibilities of partners, making collaboration a secondary concern rather than a core and consensual strategy clearly elaborated on the basis of a number of consultations/discussions, with the strong commitment of all partners. This is consistent with the fact that very little consultation around the policy occurred outside the mental health sector.

#### Integration and consistency with the national policy and legislative environments

The 1997 South Africa mental health policy guidelines, while officially not recognized at national level by the current government, has substantially influenced the direction of mental health service development in the country over the past 13 years by informing the content of provincial mental health plans, recently enacted mental health law in South Africa, and the current draft national mental health policy. While people with disabilities are included among vulnerable groups highlighted for attention in South African laws, policies and strategies, and inclusion of people with mental disabilities is specifically mentioned in the Bill of Rights, the inclusion of people with mental health conditions in provisions of other laws, policies and strategies are not consistently nor explicitly mentioned.

In Uganda and Zambia, mental health has been included in the basic health care package, consistent with the policy direction of integration stated in the two respective national mental health policies. This provides evidence of these governments' commitment to integrate mental health into the overall health system. However there are no development and social welfare policies in these countries within which to integrate the care and rehabilitation needs of people with mental health conditions, nor have their needs been integrated into the poverty alleviation strategies of these countries.

In Ghana, the 1994 mental health policy contains concepts which are at odds with the more restrictive policy directions being promoted by the current mental health law, the NRC Decree of 1972 [20]. There is apparently little collaboration between mental health and poverty reduction or development programmes but a strong role for social welfare in the treatment and rehabilitation of patients in Ghana.

#### Overall structure, coherence and consistency of the policy content

Structures of policy documents vary widely between countries but none of the four countries included in its policy the basic key structural elements listed in the WHO Policy Checklist: a clear vision statement associated with consistent values and principles, a clear list of objectives and a detailed description of areas for action. Key values when presented were not always well articulated or organized in the document. In the case of South Africa, the lack of a clear vision statement and objectives reinforced the identity of the document as being general guidelines rather than an official policy.

Although in both the Ghanaian and the Zambian documents the objectives were clearly spelt out, they were not always consistent with the other key policy elements (vision, values and principles).

## Discussion

The analysis revealed recurrent gaps in all the policies. Six gaps identified are important enough to raise concerns over the likelihood of these policies having a significant positive effect on the countries' mental health system. These concerns, described below, represent key barriers to effective policy development which are consistent with WHO's extensive experience working with LMICs on policy and service development, in Africa and elsewhere.

- **Lack of internal consistency, both in terms of structure and of policy content**: None of the countries examined in this study satisfactorily incorporated all or a sufficient number of key policy elements (vision, values and principles, objectives, and areas for action). There were inconsistencies and even at times contradictions between the different elements. Areas for action, which should be the substance of the policy documents, were often loosely elaborated and fragmented throughout the policy document. Inconsistency reduces the strength of the document and introduces ambiguity and uncertainty over the main policy directions. It may reflect lack of commitment of those developing the policy, a degree of superficiality in some of the values being promoted, or a lack of consensus around clear directions, the focus being the production of a policy document at a particular moment rather than achieving true consensus, progressively built overtime through thorough and broad consultations between key stakeholders within the country. These deficiencies may also be related to a more overarching concern, namely a poor understanding of the role and purpose of policy making, and the lack of technical policy development skills amongst policy makers as revealed by semi-structured interviews in South Africa [21].

- **Superficiality of key (international) concepts and the predominance of a 'politically correct' discourse over real political commitment to change**: Important key international standards, such as human rights protection and promotion, users' empowerment/involvement, or evidence-based approach, were mentioned in policy documents as objectives, underlying values and principles or areas for action. However, these concepts tended to remain at a superficial level without being elaborated in a meaningful action oriented way, giving the feeling of a politically correct discourse not backed up by real commitment to implement these standards. This may reveal a discrepancy between internationally agreed upon standards of best practice and actual ideology of national policy-makers, these standards not being really perceived as essential policy directions in the countries. Again, it may also simply reveal the lack of technical skills of those involved in policy-making who, while committed to international standards and good practices were not clear on how to elaborate them into concrete, operational policy directions and strategies.

- **Lack of evidence and data on which to base decisions on policy directions**: For clear decisions to be made, basic information on the existing situation is necessary. Two of the four countries specifically mention the need for epidemiological and other population based intervention studies to inform policy and service planning and development. The need for accurate information systems for mental health is absent in three of the policies, although this is identified as a key need by stakeholders interviewed in all 4 countries [8]. Situational analysis to inform policy development was not sufficiently elaborated in three of four countries, raising concerns on how adequately the policy could really meet the mental health needs of the country and on which basis monitoring and evaluation can be designed. This can also explain the lack of precision in the description of policy actions, for example different aspects of human resources and training or medicines procurement and distribution. The lack of precision is particularly worrying if we consider that none of the four countries had a plan associated with the mental health policy being evaluated. However, having accurate information is not sufficient. Despite the availability of a comprehensive, documented situational analysis, the Zambian policy, for example, does not sufficiently address the key issues requiring attention in the country. Proper use of research to inform policy development requires the development of skills to translate research into policy directions and objectives. Similarly it is not sufficient to simply put in place information systems that collect accurate and timely data without developing the capacity of programme managers, planners and policy makers to use the information in a way that facilitates the monitoring, evaluation and improvement of the policy and plan implementation process.

None of the countries had a built-in process to evaluate the development and implementation of their policy and plan despite these being crucial steps for the development of a comprehensive document achieved through an inclusive process.

Researchers can also support policy development by ensuring that the evidence they generate is made accessible to policy makers and planners, as has been the case with two seminal reports on mental health which clearly and accessibly present sound evidence to inform directions for mental health policy, service development and resourcing, namely the World Health Report 2001, and the newly launched Mental Health and Development Report, 2010 [5,22].

- **Inadequate political support**: The checklist assessment also revealed an overall lack of high level political support from the Ministry of Health in some instances, and in others, lack of support from key stakeholder groups outside of the health sector, with most of them not having been consulted during policy development. Strong support and active participation in policy development from the Ministry of Health and cabinet are essential, first to ensure more consistency of the mental health policy with other current government policies, and second to obtain formal approval and governmental support in implementation. Too often the policy drafting group is restricted to a group of mental health specialists and academics who concentrate their efforts in putting together a 'technically sound' policy document, entirely based on their clinical and/or research experience. Science and personal experience are not valuable enough arguments 'per se' for a government to adopt a policy document, and this approach might result in a shelved policy document which has little if any impact on improving health care in the country. Policy development also requires strong support and participation from health and mental health care professionals who will be the 'first line' implementers. However, consultations need to be broader than the mental health and the general health sectors if a comprehensive mental health policy promoting integration into the health system and community life (including access to education and employment) is to become a reality [23]. Support, awareness and ownership at all levels are needed for dissemination and implementation to be successful. Users of services and families need to be an important part of the consultation process because these are the groups who are meant to benefit from services [24]. However, consultations in countries did not meet these basic requirements. Political support was lacking because it was not sufficiently and actively built prior to and during policy development. For users and families to be involved, for example, empowerment, collaboration and partnerships must be built beforehand [25]. This explains the unsuccessful attempt in South Africa to get the Federation for Mental Health to provide users' inputs to the policy development process. A mental health policy, to be successfully implemented, requires support from all levels in the country, from users to governments.

- **Poor integration of mental health policies in the overall national policy and legislative framework**: Notably, when there is a degree of consistency and integration of key policy elements into the broader health or development policy of a country the implementation of the mental health policy is more likely to be successful. Integration into the broader development policy allows for a less prioritized programme such as mental health to benefit from the attention given to the development agenda. By optimizing available resources, integration increases the likelihood of mental health activities being implemented (e.g. training of health and social workers in mental health, provision of services), monitored and evaluated (e.g. collection of mental health indicators within health information systems). An example is in the case of Uganda where mental health had been included in the basic health care package and was part of capacity building of health workers at primary care level [26].

- **Lack of specificity for financing the mental health policy**: Adequate financing is the lifeblood of effective implementation of a mental health policy. Policy development should include activities designed to identify available sources of funds within the health budget and to promote intersectoral collaboration within the budgets of other sectors responsible for mental health related policy actions. Feasible mechanisms for the allocation and monitoring of funds to effect actions prioritised in the mental health policy should be identified and specified in the policy to ensure that implementers have the necessary funds for implementation once the policy is approved. Pilot projects should be designed to assess whether the planned actions specified within the policy can indeed be implemented with available resources, and to identify missed opportunities for financing policy actions when these are scaled up [27,11].

### What can be done?

When looking at ways to address the key structural concerns described above, three major strategies emerge: First of all, the capacity of key stakeholders, including policy-makers, in public (mental) health and policy development must be strengthened. *Technical *policy skills of policy-makers can be improved through a number of strategies (e.g. in-service and outsourced training courses and workshops, post-graduate learning opportunities, peer exchanges and mentoring). In addition, opportunities must be created for members of other key stakeholder groups (users, health professionals, social workers, etc) to develop and improve their policy skills if they are to significantly participate in policy development in their country. Second, a new culture of dynamic policy development must be created. Policy making must be understood as a cyclical, inclusive and dynamic process rather than a momentary snapshot in the story of health development in the country. Policies reviewed in this study did not reflect some of the values, principles and practices regarded as standard best practice today, reflecting the impact of the effect of the spirit of the times on policy formulation. The vision, key values and objectives for mental health in the country as described in the policy document must be built with all concerned groups, monitored, evaluated, questioned and redesigned consensually in an ongoing process in order to remain adapted to the level of needs and resources. Policy making must be an inclusive process, blending very diverse experiences and expertises into a national consensus, rather than based on the vision of a very restricted group of experts regarding what mental health care should be. This requires addressing power issues around roles and responsibilities for mental health in the country [23]. Further, policy must be responsive and constantly monitored in order to ensure that the real needs of the population served are addressed. Finally, action must be coordinated, both within mental health and beyond, in order to optimize resources available. Good governance is essential to bring about effective coordination of actions in the field of health and mental health, and between health and other sectors, to ensure that both overlaps and gaps in service development and delivery are minimized in pursuit of better health outcomes and overall development.

### Limitations

The current comparative analysis is subject to the bias of any qualitative research led in the policy area. The quality of data collected is tied to the thoroughness of comments made by reviewers, their subjectivity and their broader capacity of interpretation of the policy context. Levels of consultation and participation in the review process between each country differed: Ghana and Uganda had a large consultation process with a number of stakeholders whereas South Africa included only the research team and former Director of Mental Health and Substance Abuse. Adding to the subjectivity, the process section requires inputs from people having witnessed, and most often been personally involved in the policy development. South African reviewers, for example, though a small group, included individuals who possessed a high level of historical and institutional knowledge about the policy development process for the policy reviewed. Any bias in the assessment was minimized through the methodology including the use of a comprehensive and detailed WHO tool, the engagement of a wide range of key stakeholders for the content and process analysis, and the independent assessments by the WHO team who had distance from national processes. In addition the WHO team returned to interview key national stakeholders whenever the information was insufficient or showed any discrepancies. The interpretation of the results of the WHO checklist has been supported in this study by the results of other tools of policy analysis used within the MHaPP project (e.g. prior knowledge of key actors, semi-structured interviews, WHO-AIMS) [7,20,28].

## Conclusion

This paper, through the assessment of four mental health policies developed in LMICs, highlights a number of important gaps in policy making for mental health which are likely to impede the development of good quality services and supports. It also identifies skills, power, and coordination as three key challenges to good policy making. The findings also underscore the need for policy makers to realise the concepts of participation and respect for human rights in the formulation of policies and the necessity for routine revision of policy in keeping with the time.

## Competing interests

The authors declare that they have no competing interests.

## Authors' contributions

EF was involved in the conception & design, analysis & interpretation stages of the assessment as well as drafting and critical revisions of the manuscript. MF contributed to conception & design, analysis & interpretation, drafting and critical revisions of the manuscript. SK was involved in acquisition of data, analysis & interpretation, drafting and critical revisions of the manuscript. AO participated in conception & design, analysis & interpretation and critical revisions of the manuscript. JS was involved in acquisition of data, analysis & interpretation, and critical revisions of the manuscript. JM participated in acquisition of data, analysis & interpretation, and critical revisions of the manuscript. CK contributed to analysis & interpretation and drafting of the manuscript. AF was involved in conception and design phases of the study and critical revisions of the manuscript. MF, SK, AO, JS, and JM approved the final manuscript.

## Author information

EF, MF, AO and AF are principal investigators/lead partners on the Mental Health and Poverty Project, and SK, JS and JM are country level research officers on the study. CK was a consultant for WHO during the drafting of the paper.
